# A global perspective of antibiotic-resistant *Listeria monocytogenes* prevalence in assorted ready to eat foods: A systematic review

**DOI:** 10.14202/vetworld.2021.2219-2229

**Published:** 2021-08-26

**Authors:** Prudence Mpundu, Allan Rabson Mbewe, John Bwalya Muma, Wizaso Mwasinga, Nawa Mukumbuta, Musso Munyeme

**Affiliations:** 1 Ministry of Health, Levy Mwanawasa Medical University, Lusaka 10101, Zambia; 2 Department of Disease Control, School of Veterinary Medicine, University of Zambia, Lusaka 10101, Zambia; 3 Department of Environmental Health, School of Public Health, University of Zambia, Lusaka, Zambia; 4 Department of Epidemiology and Biostatics, Levy Mwanawasa Medical University, Lusaka, Zambia

**Keywords:** antibiotic resistance, *Listeria monocytogenes*, ready-to-eat foods

## Abstract

**Background and Aim:**

*Listeria monocytogenes* in ready-to-eat (RTE) foods remains consistently under-reported globally. Nevertheless, several independent studies conducted to investigate have elucidated the prevalence and antibiotic resistance profiles of *L. monocytogenes* in RTE-associated foods and their antibiotic resistance profiles. Given the rapid increase in consumption of RTE foods of both animal and plant origin, it is imperative to know the prevalence deductive data focusing on how much of *L. monocytogenes* is present in RTE foods, which is critical for food safety managers and retailers to assess the possible risk posed to end-users. In addition, valuable insight and another angle to the depth of the problem, we conducted a systematic review and meta-analysis to synthesize available data regarding the prevalence of *L. monocytogenes* in RTE foods and antibiotic resistance profiles.

**Materials and Methods:**

We conducted a meta-analysis study of *L. monocytogenes* and antibiotic resistance to clinically relevant antibiotics to determine the extent of *L. monocytogenes* contamination in RTE foods and antibiotic resistance profiles. The primary search terms, also known as keywords used, were restricted to peer-reviewed and review articles, and databases, including Google Scholars, Science-Direct, and Scopus, were searched. The inclusion of articles meeting eligibility criteria published between 2010 and 2020 after title, abstract, and full article screening. Data analysis was performed at multiple stages using quantitative meta-analysis reviews.

**Results:**

*L. monocytogenes* pooled proportion/prevalence was highest in chicken products determined at (22%) followed by various but uncategorized RTE foods at 21%. Regarding antibiotic resistance, profiling’s highest pooled prevalence resistance was observed in penicillin at 80% resistance, followed by cephalosporin at 47%.

**Conclusion:**

Within its limitations, this study has attempted to provide insight into the pooled proportion/prevalence of *L. monocytogenes* in RTE foods and the antibiotic resistance profile at the global level. Determining the proportion/prevalence of *L. monocytogenes* in RTE foods across the globe and antibiotic resistance profile is essential for providing quality food and reducing public health problems due to unsuccessful treatment of foodborne illness. This study provides insight into the pooled prevalence of L. monocytogenes in RTE foods and the antibiotic resistance profile. The results of this study partly endeavored to help appropriate authorities strengthen their preventive measures on specific RTE foods that are most likely to be contaminated with *L. monocytogenes* and antibiotic resistance profiles.

## Introduction

*Listeria monocytogenes* Gram-positive ubiquitous bacterium known to cause listeriosis, recognized as a foodborne pathogen in the early 1980s [1]. It is a known psychotropic microorganism and can survive in harsh environmental conditions [2]. However, most pathogens, including *L. monocytogenes*, can be killed by pasteurization temperatures [3]. Unlike most other foodborne pathogens, *L. monocytogenes* can grow in food with reasonably low moisture content, at refrigeration temperature and high salt concentrations, especially in ready to eat (RTE) foods in contrast with many other foodborne pathogens [4]. It is difficult to measure the global impact of listeriosis infections in assorted foods because the incubation period can be prolonged [5]. Listeriosis is rarely described and is under-reported due to inadequate linkage between the food consumed and the subsequent infection; most cases are sporadic, except for food producers who conduct quality assurance activities [1,6].

The World Health Organization (WHO) annual report of 2015 indicated that 600 million people fall ill globally due to foodborne diseases [7]. South-East Asia accounts for the highest global burden of foodborne diseases per population among the WHO regions, with global infection and deaths of about 150 million and 175 thousand, respectively [8]. On the other hand, Africa accounts for about 91 million people with foodborne diseases [7]. The United States preliminary report done in 2019 showed increased pathogens such as *Listeria* transmitted through food. However, the burden remained the same, indicating an impossibility to meeting the set target of “Healthy people 2020” by reducing foodborne illness [9]. RTE chicken samples in United Kingdom between 2016 and 2017 were analyzed, where an association by the authors regarding the source of human listeriosis was established [10]. A report done in the European Union (EU) indicated that despite the application of food safety criteria for *L. monocytogenes* in RTE foods, no significant decrease from their findings was recorded in human invasive listeriosis [11]. The significant increasing trend of listeriosis was observed during 2011-2015 by the EU member states from 1516 cases in 2011 to 2,242 2015 [12]. While a European Food Safety Authority report concluded that fish (10.4%), meat (2.1%) and cheese (0.5%) as foods that had the highest frequency of contamination of RTE from the whole RTE foods sampled [13].

Most importantly, listeriosis is a relatively rare illness compared to other foodborne infections; it is under-reported because surveillance activities are lacking [14]. According to available prevalence studies, approximately 5.1% of dairy and RTE food samples were contaminated with *L. monocytogenes* [14]. Cases of listeriosis caused by consumption of RTE foods have been in existence for years in Africa, as indicated in earlier studies [14], the 2019 outbreak in South Africa and a meta-analysis article conducted in Africa on foodborne pathogens [15,16]. In addition, food contamination has been evidenced by literature is known to be one of the principal vehicles of *L. monocytogenes* infections, especially in RTE foods, because of the absence of a heating step before consumption [17].

*L. monocytogenes* is resistant to some commercially available antibiotics and sanitizers [18]. Antibiotic resistance is increasingly becoming diverse [19] due to increased antibiotic usage [20]. Resistance rates in some European countries exceed 40-50%; moreover, antibiotic resistance also contributes to the unsuccessful treatment of infections, leading to more severe and prolonged illnesses [21]. Furthermore, this causes production reduction, which affects livelihoods, including food security. In addition, the increase in global trade and travel necessitates the spread of antimicrobial resistance between countries and continents. The United Nations, in September 2016, indicated a global rise in antimicrobial resistance as a threat to health and human development [21].

Other authors have done meta-analysis and systematic reviews on this subject; a gap still exists regarding the estimates of the prevalence of *L. monocytogenes* in RTE foods and their antibiotic resistance profile in RTE foods [22,23]. According to the WHO, listeriosis is one of the most important zoonotic diseases globally [7,15,24]. Therefore, knowing the antibiotic-resistant *L. monocytogenes* in various RTE foods is cardinal in treating listeriosis in humans [25]. Furthermore, this may help guide policy on prudent use of antibiotics, especially those administered both in humans and animals [26].

This meta-analysis aimed to bring out the global perspective of antibiotic-resistant *L. monocytogenes* prevalence in assorted RTE foods by synthesizing published data. It also aimed to identify the RTE foods that are more likely to be contaminated with *L. monocytogenes* and the antibiotic resistance profiles in the different RTE foods.

## Materials and Methods

### Ethical approval

This is a meta-analysis study, and it does not require ethical approval.

### Study period and location

This systematic/desk review was done in Zambia, Lusaka; however, the literature search on published data was based on citations from studies conducted from January 2010 to October 2020. The data analysis was conducted using Stata Version 15 (Stata Corporation, TX, USA).

### Search strategy

**Figure-1:**
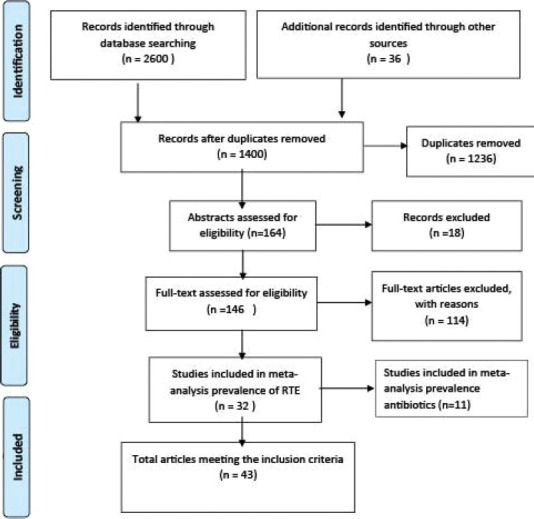
The flow diagram of the literature search and selection of eligible studies.

We used Cochrane protocols to conduct a meta-analysis [27]. According to the PRISMA protocols, we selected and extracted them (Figure-1) [28,29]. Searching literature involved restricting peer-reviewed published articles in PubMed, Science Direct, Google Scholar, and Scopus on papers reporting *L. monocytogenes* in RTE foods and antibiotic-resistant profiles from January 1, 2010, to October 2020. Keywords such as: “prevalence” or “occurrence,” or “Bacteria,” “*Listeria*,” “*L. monocytogenes* in RTE foods,” and “antibiotic resistance in RTE foods” were used to search the databases. The extracted articles formed a reference list that the authors used for further screening to obtain additional relevant publications.

### Inclusion/exclusion criteria

Criteria used for the inclusion of the article: 1. English –language full text; 2. cross-sectional and descriptive studies; 3. articles with known sample size; and studies reported the prevalence of either RTE foods or antibiotic resistance or reported both; 4 studies reported both RTE foods and raw foods and five studies that reported other bacteria, including *L. monocytogenes*. The authors excluded all books, workshop, and thesis due to a lack of peer review [30].

### Data extraction

Data extracted from all considered articles included: the year of study, author name, country, data of the study, name of RTE foods, name of the antibiotic, total sample size, positive sample size, sample source, sampling technique, and method of isolation.

### Meta-analysis data

Data were entered first in the Microsoft Excel spreadsheet for collation and cleaning and later exported into Stata software (Stata Corporation) for data analysis. Studies found were summarized using counts in a flow chart, and then cross-tabulations between food types and antibiotic resistance were performed to check for patterns. Meta-analysis was performed on all eligible studies to establish the global pooled prevalence of *L. monocytogenes* contamination of RTE foods and the associated antibiotic resistance profiles. The stratification of the meta-analysis was according to the type of RTE food, the country where the study originated, and the types of antibiotics used. Food grouping active types, such as fish, beef, chicken, and dairy RTE products, reduces the differences that may arise due to heterogeneity in the meta-analysis. We used the I^2^ to determine the heterogeneity of the studies, and if the I^2^ was >50%, heterogeneity was considered [31].

## Results

### Literature search

As depicted in Figure-1, a total of 2636 articles were identified from four peer-reviewed electronic databases: PubMed (388), Science Direct (43), Google Scholar (2162), and Scopus (38) published electronically dated back from January 2010 to October 2020. These 1236 duplicates identified were removed using EndNote 9× referencing software (Philadelphia, PA, USA). Thus, a total number of 164 articles remained after excluding redundant articles, of which 18 more were excluded due to lack of relevance of their title, not falling under any stipulated inclusion criteria stated by the authors. In addition, we further revised the full text of the 146 remaining articles. Furthermore, we further excluded 114 articles due to a lack of essential data, such as positive sample size or total sample size. Finally, only 32 articles reported the prevalence of *L. monocytogenes* and 11 articles reported on both the prevalence of *L. monocytogenes* and antibiotic profiles of *L. monocytogenes* considered appropriate for this meta-analysis, making the total number of studies to be 43.

### Distribution of the type of RTE foods across studies

Table-1 shows the number of publications from the continents that we included in this meta-analysis. Europe and Asia had more papers (n=15 and 12, respectively) than other continents, while Africa had 7, with North and South America reporting two papers each.

**Table-1:** Number of publications reviewed by continent and the type of foods reported.

Food Type	Number of publications continents					

	Europe (n=15)	Asia (n=12)	Africa (n=7)	North America (n=2)	South America (n=2)	Total
Beef products	8	8	-	-	16
Milk	7	2	5	-	-	14
Fish	14	8	2	-	-	24
Salads	9	4	2	-	2	17
Pork	4	-	-	-	-	4
Vegetable product	2	4	1	4	-	11
Chicken product	1	9	5	-	-	15
Assorted ready-to-eat foods	7	8	3	2	-	20
Total	52	43	18	6	2	121

The food products differed in terms of their origin, type, and risk of *L. monocytogenes* contamination. Subsequently, we grouped these food items into a broader range of commodities for analysis. Most of the studies in Europe reported on Fish products, RTE salads, beef products and assorted RTE foods, while in Asia, the highest reported RTE foods were chicken products, beef products, assorted RTE foods, and fish products. While in Africa, the food with the highest frequency of reporting was chicken products and milk products, while North and South America had vegetable products and salads (Table-1).

### Distribution of antibiotics resistance across different types of RTE foods

We studied various antibiotics for resistance in different RTE foods, and antibiotics we grouped into different classes that were homogeneous. The total number of antibiotics reported after grouping was 13, while the number of food types was 4. The antibiotics reported with many papers were penicillins, quinolone, aminoglycosides, lincomycins, and macrolides. The foods reported exhibiting resistance to antibiotics were assorted RTE foods and fish products (Table-2).

**Table-2:** Distribution of antibiotics resistance across different types of ready-to-eat foods.

Antibiotics	Type of food				

	Assorted foods	Poultry products	Ready-to-eat salads	Fish product	Total
Aminoglycosides	12	2	5	0	19
Antimycobacterials	4	1	1	0	6
Carbapenem	-	0	0	1	1
Cephalosporin’s	10	0	0	2	12
Fluoroquinolones	1	0	1	0	2
Glycopeptide	7	1	0	1	9
Lincomycin	3	0	1	2	6
Macrolides	6	1	2	1	10
Nitrofurantoin	1	0	0	0	1
Oxytetracycline	1	0	0	0	1
Penicillins	20	3	2	7	32
Quinolone	12	2	2	4	20
Sulfonamides	5	1	0	1	7
Tetracycline	9	1	2	3	15
Total	91	12	16	22	141

### Country prevalence of *L. monocytogenes* contamination RTE foods and global pooled prevalence

The global pooled prevalence contamination of RTE foods with *L. monocytogenes* was 10.8% (95% CI 9.4-12.2). The prevalence of *L. monocytogenes* contamination in RTE varied by country, from 4.5% in Estonia Eastern Europe to 56.3% in Jordan Middle Eastern Asia. The heterogeneity across the studies was high at 71.1. However, the within-country heterogeneity ranged from homogeneous 0.0 in Italy and other countries to very high at 91.9 in Sweden. Table-3 summarizes prevalence, weights, and heterogeneity in studies by country and global pooled prevalence.

**Table-3:** Country prevalence of *Listeria monocytogenes* contamination ready-to-eat foods and global pooled prevalence.

Country	No. of studies	ES^a^	Lower	Upper	Weight	Heterogeneity	Degrees of freedom	p-value	I^2^(%)d
Spain	3	0.090	0.045	0.131	11.52	19.19	7	0.008	63.5
Italy	2	0.271	0.194	0.347	3.31	3.28	4	0.512	0.0
Japan	2	0.063	0.004	0.121	5.76	0.84	10	1.000	0.0
Turkey	2	0.110	−0.045	0.268	0.78	0.10	3	0.992	0.0
Sebia	1	0.251	0.146	0.355	1.78	1.72	1	0.190	41.7
Northern Ireland	1	0.084	−0.010	0.178	2.20	0.82	2	0.664	0.0
Sweden	1	0.232	0.175	0.289	6.01	49.22	4	0.000	91.9
Greece	2	0.081	−0.055	0.217	1.05	0.46	4	0.977	0.0
Estonia	1	0.045	0.022	0.67	38.96	44.82	7	0.000	84.4
Iran	3	0.276	0.186	0.365	2.44	48.56	5	0.000	89.7
Egypt	1	0.233	−0.010	0.476	0.33	0.64	1	0.423	0.0
India	1	0.052	−0.225	0.329	0.25	0.02	1	0.886	0.0
Algeria	1	0.063	−0.114	0.240	0.62	0.12	1	0.732	0.0
Ethiopia	1	0.089	−0.059	0.237	0.89	0.24	3	0.971	0.0
United Kingdom	3	0.133	0.075	0.191	5.85	11.58	5	0.041	56.8
Malaysia	5	0.139	0.079	0.199	5.48	12.64	18	0.812	0.0
Brazil	2	0.176	0.0	0.049	0.304	1.20	21.61	0.001	76.9
Nigeria	2	0.241	0.159	0.322	2.97	23.66	3	0.000	87.3
China	5	0.053	−0.012	0.118	4.58	1.31	6	0.971	0.0
Thailand	1	0.147	0.022	0.273	1.24	0.33	1	0.563	0.0
Jordon	1	0.563	0.426	0.699	1.06	14.82	2	0.001	86.5
Sudan	2	0.124	0.018	0.230	1.74	1.20	4	0.879	0.0
Overall	43	0.108	0.094	0.122	100.0	407.82	118	<0.000	71.1

### The global pooled prevalence of antibiotic resistance of *L. monocytogenes* in RTE foods

The global pooled prevalence of antibiotic-resistant *L. monocytogenes* in RTE foods was 38.1% (95% CI 36.1-39.7). The prevalence ranged from 9% in Taiwan to 94.1% in South Korea. The heterogeneity in the studies was very high at 89.9. Table-4 summarizes the prevalence of antibiotic resistance in *L. monocytogenes* in RTE foods.

**Table-4:** The global pooled prevalence of antibiotic resistance of *Listeria monocytogenes* in ready-to-eat foods.

Country	No. of studies	ES^a^	Lower	Upper	Weight of ES (%) ^b^	Heterogeneity statistic	Degrees of freedom	p-value	I^2^(%)^d^
Brazil	1	0.250	0.005	0.495	0.42	0.00	1	1.000	0.0
China	5	0.146	0.115	0.178	26.24	201.7	37	0.000	81.7
Ethiopia	1	0.487	0.344	0.631	1.24	5.80	3	0.122	48.3
Iran	2	0.267	0.225	0.308	14.78	53.57	18	0.000	66.4
Jordan	1	0.121	−0.137	0.378	0.38	0.13	2	0.936	0.0
Korea	1	0.941	0.883	1.00	7.44	24.52	1	0.000	95.9
Malaysia	1	0.802	0.696	0.908	2.27	7.32	2	0.026	72.7
Nigeria	2	0.469	0.441	0.496	33.62	120.67	19	0.000	84.3
Spain	1	0.164	0.004	0.324	0.99	0.59	6	0.997	0.0
Taiwan	1	0.091	−0.306	0.488	0.16	0.00	0	-	-
Turkey	4	0.448	0.392	0.504	8.04	142.60	24	0.000	83.2
Overall	20	0.381	0.365	0.397	100.0	1342.08	136	0.000	89.9

Effect size (ES): Prevalence of antibiotic resistance in ready-to-eat foods (ratio of positive samples/total samples). The weight of ES is related to the total sample size of individual studies. Variation in study outcomes between studies. Heterogeneity across studies (I^2^)

### Prevalence of *L. monocytogenes* contamination in RTE foods

In terms of specific food products, *L. monocytogenes* contamination ranking was as follows: The highest was in chicken products at 22% (95% CI 0-28), followed by milk products at 18% (95% CI 9-27) and the smallest amount of contamination was found in fish and pork products at 12% (95% CI 9-15) and 8% (95% CI 12-20), respectively. Table-5 summarizes the prevalence of *L. monocytogenes* contamination in specific RTE foods.

**Table-5:** Prevalence of *Listeria monocytogenes* contamination in ready-to-eat foods.

Item	Country	No. of Papers	Effect size	95% CI	Weight	I Squared (%)	p-value
	Spain	2	0.25	0.08, 0.43	27.61	86.6	<0.001
Milk products	Italy	1	0.02	0.52, 0.56	2.75	0	<0.001
	Sweden	1	0.36	0.29, 1.05	1.79	0	<0.001
	Estonia	2	0.21	0.07, 0.35	41.28	0	0.578
	India	2	0.05	0.23, 0.33	10.6	0	0.886
	Ethiopia	2	0.07	0.15, 0.30	15.97	0	0.678
	Overall	10	0.18	0.09, 0.27	100	46.5	0.044
Fish products	Spain	2	0.08	0.02, 0.14	28.24	0	0.685
	Italy	1	0.34	0.20, 0.48	5.66	0	0.001
	Japan	1	0.08	−0.01, 0.14	21.13	0	0.999
	Turkey	1	0.12	−0.25, 0.49	0.8	0	0.001
	Sebia	1	0.25	0.15, 0.36	9.88	41.7	0.19
	Northern Ireland	1	0.01	−0.31, 0.34	0.01	0	0.001
	Sweden	1	0.12	0.06, 0.19	25.84	0	0.449
	Greece	1	3.34	−0.12, 0.24	3.34	0	0.923
	Estonia	1	0.06	−0.42, 0.53	0.48	0	0
	Iran	1	0.06	−0.20, 0.30	1.78	12.4	0
	Egypt	1	0.23	−0.01, 0.48	1.84	0.423	0
	Overall	12	0.12	0.09, 0.15	100.0	12.4	0.289
Pork products	Spain	1	0.08	0.03, 0.14	100	0	0.736
Ready-to-eat salads	Italy	1	0.27	0.16, 0.38	13.91	0	0
	Turkey	1	0.18	−0.35, 0.71	0.56	0	0
	Northern Ireland	1	O.O4	−0.13, 0.20	5.8	0	0
	Estonia	1	0.19	0.11, 0.26	27.69	0	0
	Iran	1	0.31	0.03, 0.59	1.96	0	0
	United Kingdom	1	0.04	−0.06, 0.13	17.13	0	0
	Malaysia	3	0.14	0.07,0.22	26.31	25.4	0.235
	Brazil	2	0.68	0.38, 098	1.76	85	0.001
	Nigeria	2	0.2	0.02, 0.38	4.89	46.6	0.171
	Overall	13	0.16	0.12, 0.20	100.0	62.4	<0.001
Beef products	Japan	1	0.07	0.21,0.35	0.58	0	0.787
	Northern Ireland	1	0.12	−0.00,0.24	3.08	0	0
	Sweden	1	0.61	0.49,0.73	3.11	0	0
	Estonia	1	0.02	−0.00,0.04	80.32	0	0
	Iran	2	0.33	0.21,0.45	3.13	95.5	0.001
	United Kingdom	1	0.02	−0.16,0.20	1.4	0.991	0.001
	Malaysia	1	0.07	−0.28,0.41	0.39	0	0
	China	4	0.05	0.03,0.12	7.75	0	0.743
	Thailand	1	0.27	−0.16,0.71	0.24	90.3	0.001
	Overall	13	0.16	0.12, 0.20	100.0	62.4	<0.001
Chicken products	Turkey	1	0.1	−0.11, 0.32	7.7	0	0
	Iran	1	0.26	0.07, 0.45	9.93	0	0
	Malaysia	1	0.22	0.05, 0.40	11.64	0	0.972
	China	2	0.07	−0.06, 0.20	20.5	0	0.965
	Jordan	1	0.56	−0.43, 0.70	18.95	86.5	0.001
	Sudan	1	0.12	−0.02, 0.23	31.28	0	0.879
	Overall	7	0.22	−0.16, 0.28	100.0	72.1	<0.001
Assorted ready-to-eat	Italy	1	0.1	−0.19,0.39	2.44	0	0
	Japan	1	0.09	−0.05, 0.24	9.85	0	0
	Greece	1	0.1	−0.10, 0.31	4.86	0	0.986
	Estonia	1	0.47	0.26, 0.67	5.09	80.7	0.023
	Algeria	1	0.14	−0.34, 0.83	0.9	0	0
	Ethiopia	1	0.1	0.10, 0.30	5.45	0	0.916
	United Kingdom	3	0.22	0.14, 0.30	33.65	0	0.411
	Malaysia	1	0.1	−0.04, 0.23	11.64	0	0.609
	Nigeria	1	0.48	0.35, 0.61	12.06	0	0
	China	1	0.06	−0.28, 0.41	1.8	0	0
	Thailand	1	0.14	0.01, 0.27	12.24	54.7	0.002
	Overall	13	0.21	0.16, 0.25	100.0	54.7	0.002

### The global pooled prevalence of contamination of RTE foods with antibiotic-resistant *L. monocytogenes*

In terms of *L. monocytogenes* contamination of RTE foods with antibiotic resistance, the highest resistance was from penicillins at 80% (95% CI 57-64), followed by cephalosporin’s at 47% (95% CI 44-56%) and the lowest antibiotic resistance was for sulfonamides and carbapenem at 13% (95% CI 3-23) 6% (95% CI 0-45), respectively. Table-6 summarizes the prevalence of antibiotic-resistant *L. monocytogenes* contamination in RTE foods.

**Table-6:** The global pooled prevalence of contamination of ready-to-eat foods with antibiotic-resistant *Listeria monocytogenes.*

Item	Country	No. of papers	Effect size	95% CI	Weight	I Squared (%)	p-value
Aminoglycosides	China	7	0.07	−0.00, 0.14	60.75	0.0	0.985
	Iran	2	0.10	−0.17, 0.28	1.77	0.0	0.855
	Italy	2	0.06	0.17, 0.28	6.27	0.0	0.855
	Jordan	1	0.06	−0.41, 0.52	1.46	0.0	<0.001
	Nigeria	2	0.26	0.11, 0.40	14.56	39.3	0.176
	Spain	1	0.21	0.25, 0.68	1.48	0.0	<0.001
	Turkey	2	0.39	0.24, 0.55	13.70	83.0	0.001
	Overall	17	0.07	−0.00, 0.14	60.75	0.0	0.985
Antimycobacterials	China	3	0.05	−0.06, 0.15	55.38	0.0	0.832
	Iran	1	0.08	−0.11, 0.27	17.84	0.0	<0.001
	Malaysia	1	0.83	0.66, 1.00	21.40	0.0	<0.001
	Turkey	1	0.47	0.12, 0.82	5.38	0.0	<0.001
	Overall	6	0.24	0.16, 0.32	100.0	91.9	0.001
Carbapenem	Turkey	1	0.06	−0.37, 0.49	100.0	0.0	< 0.001
Cephalosporin’s	China	3	0.52	0.44, 0.59	14.61	95.9	0.001
	Italy	1	0.15	−0.36 ,0.44	0.52	0.0	<0.001
	Nigeria	1	0.47	0.44, 0.50	82.99	0.0	0.990
	Spain	1	0.21	−0.25, 0.67	0.39	0.0	<0.001
	Turkey	1	0.15	−0.08, 0.39	1.49	0.0	0.678
	Overall	7	0.47	0.44, 0.50	100.0	88.3	0.001
Fluoroquinolones	Iran	2	0.23	0.11, 0.35	100.0	0.0	0.430
Glycopeptide	China	2	0.02	−0.13, 0.16	33.37	0.0	0.925
	Iran	1	0.35	0.19, 0.51	27.68	0.0	<0.001
	Italy	1	0.06	−0.15, 0.27	15.36	0.0	0.908
	Nigeria	1	0.57	0.23 ,0.91	5.87	0.0	<0.001
	Spain	1	0.21	−0.25, 0.67	3.25	0.0	<0.001
	Turkey	2	0.49	0.27, 0.71	14.46	67.1	0.081
	Overall	8	0.22	0.14, 0.31	100.0	68.2	0.001
Lincomycins	China	1	0.34	0.16, 0.52	28.49	0.0	< 0.001)
	Iran	2	0.06	−0.07, 0.20	49.77	0.0	0.761
	Italy	1	0.28	0.02, 0.53	13.53	0.0	<0.001
	Korea	1	0.28	0.02, 0.53	0.0	0.0	<0.001
	Turkey	2	0.13	−0.20, 0.47	8.21	0.0	<0.001
	Overall	7	0.17	0.08, 0.27	100.0	39.9	0.001
Penicillins	China	3	0.05	−0.03, 0.12	17.78	−0.0	0.981
	Ethiopia	1	0.87	0.44, 0.90	2.07	0.0	<0.001
	Iran	2	0.30	0.31, 0.48	15.12	−0.0	0.435
	Italy	1	0.47	0.36, 0.50	8.60	−88.8	0.001
	Korea	1	0.97	0.911,1.3	31.08	0.0	<0.001
	Malaysia	1	0.97	0.91, 1,03	0.0	0.0	<0.001
	Nigeria	2	0.88	0.78, 0.90	10.45	−0.0	0.58
	Spain	1	0.07	−0.25, 0.37	1.24	0.0	<0.001
	Taiwan	1	0.09	−0.31, 0.49	0.70	0.0	<0.001
	Turkey	3	0.89	0.50, 0.78	11.58	−93.5	<0.001
	Overall	16	0.80	0.57, 0.64	100.0	94.9	0.001
Quinolones	China	3	0.09	−0.01, 0.18	33.37	0.0	0.771
	Ethiopia	1	0.37	0.15, 0.60	5.74	50.2	0.156
	Iran	2	0.18	0.08, 0.29	27.00	41.3	0.182
	Nigeria	2	0.20	0.08, 0.31	21.46	29.6	0.234
	Spain	1	0.21	−0.25, 0.68	1.35	0.0	<0.001
	Turkey	3	0.41	0.25, 0.57	11.07	49.5	0.008
	Overall	12	0.19	0.14, 0.24	100.0	49.5	0.008
Sulfonamides	China	2	0.04	−0.08, 0.15	73.00	0.0	0.976
	Malaysia	1	0.39	0.07, 0.71)	9.39	0.0	< 0.001
	Nigeria	1	0.57	−0.23, 0.91	8.09	0.0	<0.001
	Spain	1	0.21	−0.25, 0.68	4.42	0.0	<0.001
	Turkey	1	0.81	−0.25, 0.61	5.10	0.0	<0.001
	Overall	6	0.13	0.03, 0.23	100.0	48.5	0.070
Tetracycline’s	China	4	0.09	−0.02, 0.19	29.93	0.0	0.810
	Ethiopia	1	0.38	0.06, 0.69	3.20	0.0	<0.001
	Iran	2	0.22	0.10, 0.34	21.17	82.7	0.016
	Italy	2	0.04	−0.19, 0.26	6.50	0.0	0.913
	Jordan	1	0.12	−0.33, 0.56	1.60	0.0	<0.001
	Korea	1	0.18	−0.13, 0.49	1.60	0.0	<0.001
	Malaysia	1	0.87	0.72, 1.02	14.80	0.0	<0.001
	Nigeria	1	0.71	0.43, 1.00	4.05	0.0	<0.001
	Turkey	2	0.30	0.14, 0.46	12.69	15.4	0.277
	Overall	15	0.30	0.24, 0.35	100.0	84.8	0.001

### Methods for isolating *L. monocytogenes* and antibiotic resistance

Most studies reviewed in this meta-analysis reported the use of internationally recognized isolation protocols recommended for the isolation of *L. monocytogenes*, International Standard Organization (70%), culture and biochemical/polymerase chain reaction (PCR) (14.1%), two-step enrichment (12.6%), and conventional methods (3.4%).

Although antibiotic resistance was determined using several methods, the majority utilized disk diffusion on Mueller-Hinton agar (39%), PCR (30%), Kirby-Bauer method (21.5%), pulsed-field gel electrophoresis method (4.7%), and the disk diffusion method, which followed the Clinical and Laboratory Standards Institute with no specifications of agar used (3.4%) and the least was the micro-broth dilution method (2%).

## Discussion

We synthesized the prevalence of *L. monocytogenes* in RTE food and antibiotic-resistant profiles globally using peer-reviewed published data in this systematic review and meta-analysis. Based on the meta-analysis results, the overall global pooled estimate of *L. monocytogenes* prevalence in RTE foods was 10.8%. In comparison, the global pooled estimate of antibiotic resistance in RTE was 38.1% among all contaminated samples. Thus, we conducted such a review study on finding the global prevalence of *L. monocytogenes* and its antibiotic profiles in RTE foods for the 1^st^ time to the best of our knowledge. Further, this study sub-analyzed the global prevalence by type of food as well as regionally by continent. We believe this study has contributed to the body of knowledge on food safety and alerts policymakers in different continents on food contamination levels with *L. monocytogenes* in their regions and globally.

Comparatively, a meta-analysis conducted among Iranian studies revealed a much lower prevalence of 4% despite having the same number of included studies of 32 with this current review [32]. However, the variance in the pooled prevalence between the two reviews were attribute to the food types included; the review from Iran did not limit its foods to RTE foods only but also included raw foods such as milk and kinds of seafood with some assortment of RTE foods. In contrast, this current review only reported on various RTE foods. *L. monocytogenes* is a post-contaminating bacterium that has a high chance of soiling RTE foods because they undergo multiple processing stages, which are likely to increase their risk of contamination which may partly explain the discrepancy [33]. In addition, the Iranian review focused on studies done within Iran and the foods included may have some level of similarity in terms of environmental factors, which may to a greater extent, decrease the risk of *L. monocytogenes* contamination compared to this current review which had a wide range of studies that came from different environmental exposures. Luber’s work cited that the differences in environmental factors equally influence the proportion of contamination in a given setting [34]. Primarily, 70% of the studies in this review reported using internationally accepted isolation, standards that have been reported to yield high discriminatory power compared to other methods, which may have contributed to the increased rate of positivity observed in the synthesized studies [35].

*L. monocytogenes* prevalence was highest in RTE chicken products (22%) compared to the other RTE foods analyzed in this study. Study on chicken products reported to have utilized more than one enrichment broth to increase the sensitivity of the detection of *L. monocytogenes* to about 97.5-98.9% compared to the expected results of 60-70% when standard regulatory protocols are used [36]. Enrichment media used may inhibit the detection of *L. monocytogenes* because the growth rate is usually slow and below detectable levels depending on the type used [36]. *Listeria*, including *L. monocytogenes*, may exist at deficient levels in foods; therefore, sample enrichment protocols must amplify these initial populations to levels that are detectable [37]. RTE salads and beef products had the least pooled prevalence. These studies had an assortment of protocols used, and the majority did not mention the specific media used for isolation. On the other hand, some selective agents used in enrichment may inhibit the repair and detection of sublethally injured *Listeria* through processing treatments such as heating [36]. These differences in protocols may have influenced the isolation variances in other RTE foods in this review study.

Notably, the distribution of studies reported on the RTE chicken products showed some similarity in geographic settings, with more studies from Asia (5), Europe, and Africa (1). Asian countries, although others like China, are still regarded as developing countries that have considerably high technological equipment and machinery for the detection of fastidious bacteria such as *L. monocytogenes* coupled with increased surveillance of foodborne diseases. The advancement in technology may have influenced the positivity rate described above, with more contamination recorded in RTE chicken products in this current review. Most importantly, poultry products are among the most critical sources of *Listeria* spp. Especially *L. monocytogenes* [38]. Novelists from original studies done by Osaili *et al*. [34] reported a higher prevalence of *L. monocytogenes* of 33.3% in RTE poultry, while Wong *et al*. [35] reported from chicken burgers and luncheon product samples 20-13.3%. Isolation prevalence in the studies described above with this review is inferred mainly due to differences in the processing methods used on the RTE chicken product [39,40].

Another remarkable finding was the high heterogeneity among the studies that reported the Prevalence of RTE foods, ranging from 0.0 to about 91.9%. The majority of the studies included in this review reported on different types of RTE foods and publication skewness within the countries. As previously indicated by other authors, the type of food influences the risk of *L. monocytogenes* contamination [34]. We may speculate the reasons for the high prevalence of *L. monocytogenes* in this review in some RTE foods such as chicken products, assorted RTE, and milk products to have been influenced by inadequate heat treatment, poor sanitation, inadequate physical separation between the raw and cooked food areas, and cross-contamination during processing and handling. In addition, *L. monocytogenes* contamination, as indicated by the results of a study done on milk products, is likely to be lower in milk than in food products such as meat and other meat products [41]. The studies in this review, despite coming from the same continent, a number of them had geographical distribution differences; for instance, the studies that came from Asia mainly came from the Eastern part.

In contrast, in Africa majority of the studies came from the Northern part with a slight difference from studies that came from Europe but with a range of some similarities. Sample size differences in the reviewed studies also played a role in contributing to the high heterogeneity observed. Adequate sample size estimation should be considered from the initial study plan in order to ensure its validity and power[42].

This review indicated that the highest resistance in various RTE foods came from penicillins (80%) and the least in sulfonamides (13%) (Table-2). Most of the antibiotics studied belonged to the class of penicillins, cephalosporins, and tetracycline. Cephalosporin and tetracycline are third-generation antibiotics intrinsically resistant to *L. monocytogenes;* these are considered inappropriate for treatment for listeriosis in infants and pregnant women [24]. Penicillin and other antibiotics such as ampicillin and oxacillin are the most well-known active β-lactams capable of inhibiting the synthesis of the bacterial cell wall of peptidoglycan [24]. *L. monocytogenes* naturally susceptible to β –lactams and the prescribed standard antibiotic therapy for treating listeriosis includes penicillin/ampicillin combined or alone together with an aminoglycoside (gentamicin) [43]. In this current review, *L. monocytogenes* resistance was highest in β-lactam antibiotics, which is a public health concern in terms of human treatment of listeriosis.

Most of these named classes of antibiotics are susceptible to *L. monocytogenes* resistance. Furthermore, *L. monocytogenes* is naturally resistant to the currently used third and fourth generation fluoroquinolones and cephalosporin’s [25]. Over the years, there has been increasing recognition of widespread antibiotic use in agriculture and aquaculture in most countries, including the ones included in this study [44]. Processing steps during food production such as slaughtering practices, farming activities and transportation of food animals may introduce resistant bacteria into the food chain [45]. Moreover, contamination during food preparation and consumption of contaminated meat and other food products may develop antibiotic-resistant bacteria in humans. Factors contributing to the rise in antibiotic-resistant bacterial species are complex and consist of various aspects. Some factors are unavoidable and inherent, such as the ability of the bacteria to adapt rapidly to changing environmental conditions because of their short generation time and the intrinsic resistance of certain bacteria. However, some variables are human, such as the extensive use of antibiotics as a growth promoter in farming practice. A significant concern to human health is the transfer of antibiotic-resistant bacteria present in food to humans and subsequent colonizing the gastrointestinal tract [45]. Overlap of use for other purposes of human medicine has also contributed to the development of resistance to antibiotics commonly [46,47]. The use of antibiotics in the food production chain is necessary, especially in export food countries [48].

Many antibiotics used in foods are the same surrogates as antibiotics used in human therapeutics; this partly explains why high antibiotic resistance in RTE foods in this review was recorded [49]. Stonsaovapak and Boonyaratanakornkit [45] reported ampicillin and rifampicin resistance in *L. monocytogenes* isolated from RTE foods. At the same time, in Columbia, a study was conducted on various RTE foods and resistance was recorded in clindamycin, streptomycin and amikacin [46]. The resistance of *L. monocytogenes* was evident in milk products to some commonly used antibiotics such as penicillin, ampicillin, tetracycline, and gentamicin, which agrees with the findings of this study [47,48]. *L. monocytogenes* are intrinsically resistant to certain antibiotics and reported by others confirming the same [1]. The picture given from the above reported prevalence from other studies is depicting a similar resistance pattern of the antibiotics with this current review study.

Globalization and exportation of foods from one country to another have also increased production using antibiotic growth promoters [26]. Most of these countries are among the highest exporters of most foods and meet the demand for food supply antibiotics. However, several foods are likely to contain antibiotic residues, especially where regulations are lacking [44]. Many countries, particularly the EU, have made substantial efforts to reduce the overall use of antibiotics in food-producing animals by benchmarking antibiotic use at the farm level and encouraging antibiotic stewardship. As a result, some foods, such as those coming from animal sources, are more prone to antibiotic use than others [21]. However, cautious use of antibiotics is needed to ensure we met regulations in food products [44]. The other underlying factor is country variations regarding the availability of guidelines on the criteria for purchasing certain drugs such as penicillin [50]. Meanwhile, most developed countries use antibiotics as growth promoters in food animals, including feed, to boost global production; this explains the country variations recorded in the current review [26].

High heterogeneity among the studies that reported antibiotic resistance was equally observed. The differences in antibiotics studied across the studies, sample size, and the sample’s origin (country) can ascribe to the above observation [49]. Easy purchase of some antibiotics such as penicillin, compared to sulfonamides antibiotics, contributes to high levels of antibiotic resistance on some groups of antibiotics compared to others. Antibiotics prescribed widely are more prone to develop resistance with time, mainly when not used according to their prescription [44]. More importantly, there is a need for susceptibility testing before some high-priority antibiotics in food products are used to curb increased antibiotic resistance. As previously mentioned, *L. monocytogenes* are naturally resistant to most antibiotics used in human and animal foods [25].

## Conclusion

This study revealed a considerably high pooled prevalence of *L. monocytogenes* in various RTE foods, and, more specifically, the RTE foods that had the highest pooled prevalence were chicken products. While the antibiotics with the highest pooled prevalence was penicillin. Therefore, control measures need to be put in place to reduce the risk of listeriosis due to consumption of RTE foods, especially chicken products. The findings also show that RTE foods are a vital source of antibiotic-resistant *L. monocytogenes*, which is a public health concern.

## Authors’ Contributions

PM: Conceived the design, analyzed the data, and revised the work. NM: Performed the cleaning of the dataset, carried out the statistical analysis, and reviewed the manuscript. MM: Contributed to a thorough review of the manuscript and supervision. ARM: Played a significant role in the early drafting and proofreading of the manuscript. WM: Involved in the proofreading of the manuscript. JBM: Contributed to manuscript drafting and proofreading. All authors read and approved the final manuscript.
